# The SQUEEZE pilot trial: a trial to determine whether septic shock reversal is quicker in pediatric patients randomized to an early goal directed fluid-sparing strategy vs. usual care

**DOI:** 10.1186/s40814-025-01731-4

**Published:** 2025-11-28

**Authors:** Melissa J. Parker, Karen Choong, Alison Fox-Robichaud, Patricia C. Liaw, Lehana Thabane, Canadian Critical Care Trials Group and the Canadian Critical Care Translational Biology Group

**Affiliations:** 1 Division of Pediatric Critical Care, Department of Pediatrics, McMaster Children’s Hospital and McMaster University, 1280 Main St W, HSC 3E-20, Hamilton, Ontario L8S 4K1 Canada; 2 Department Health Research Methods, Evidence and Impact, McMaster University, 1200 Main St W, Hamilton, Ontario L8N 3Z5 Canada; 3 Division of Emergency Medicine, Department of Pediatrics, The Hospital for Sick Children and University of Toronto, 555 University Avenue, Toronto, Ontario M5G 1X8 Canada; 4 Department of Anesthesia, McMaster University, 1200 Main St W, Hamilton, Ontario L8N 3Z5 Canada; 5 Biostatistics Unit/FSORC, St Joseph’s Healthcare Hamilton, 3rd floor Martha Wing, 50 Charlton Avenue East, Hamilton, L8N 4A6 Canada; 6 Department of Medicine, McMaster University, DBRI, Rm C5-106 & 107, 237 Barton Street East, Hamilton, Ontario L8L 2X2 Canada; 7 Faculty of Health Sciences, University of Johannesburg, Johannesburg, South Africa

**Keywords:** Fluid therapy, Resuscitation, Shock, Sepsis, Pediatrics

## Abstract

**Objective:**

The overall objective of our research is to determine in children with septic shock whether the use of a fluid-sparing strategy results in improved clinical outcomes without an increased risk of adverse events compared to usual care. The specific objective of this pilot randomized controlled trial was to evaluate the feasibility of a definitive multicenter trial to answer our research question.

**Design:**

Pragmatic, 2-arm, parallel group, open-label, prospective pilot randomized controlled trial including a nested biosample-based translational study.

**Setting:**

Pediatric tertiary care center.

**Patients:**

Children aged 29 days to <18 years of age presenting to the emergency department or admitted to an inpatient ward (including the PICU) with suspected or confirmed septic shock and a need for ongoing resuscitation.

**Interventions:**

Fluid-sparing vs. usual care resuscitation strategy continued until shock reversal. The fluid-sparing intervention comprised instructions to restrict fluid bolus therapy in conjunction with early initiation and/or preferential use of vasoactive medication support as a strategy to spare fluid while targeting the hemodynamic goals specified in the American College of Critical Care Medicine Surviving Sepsis Guidelines. The usual care strategy did not limit the use of fluid bolus therapy.

**Measurements and main results:**

Fifty-three were randomized to usual care (*n* = 27) or fluid-sparing (*n* = 26). Fifty-one participants were available for primary outcome analysis. Primary feasibility outcomes related to participant enrollment and protocol adherence. Enrollment rate was 1.8 (51/29); 95% confidence interval [CI]: 1.3–2.3 participants/month. Study procedures were implemented in 49/51 (96.1%), 95% CI: 86.5–99.5% participants within 1 h of randomization in a median (interquartile range [IQR]) of 8 (5, 15) minutes. The protocol required the use of an exception to consent process and consent for ongoing participation was 48/51 (94.1%), 95% CI: 83.8–98.8%. There were no serious adverse events.

**Conclusions:**

We concluded the large multicenter SQUEEZE trial feasible to conduct.

**Trial registration:**

ClinicalTrials.gov [NCT01973907]. Registered October 27, 2013, https://clinicaltrials.gov/study/NCT01973907.

**Supplementary Information:**

The online version contains supplementary material available at 10.1186/s40814-025-01731-4.

## Key messages regarding feasibility

- It was uncertain whether appropriate patients could be rapidly identified, randomized, and the time-sensitive intervention quickly implemented in the context of an individual medical emergency (septic shock). Practical aspects of implementing the “exception to consent” (deferred consent) process were also untested.
- Key feasibility findings are that the eligibility criteria supported recruitment into the trial at an acceptable rate, that the intervention and control treatment strategies could be implemented quickly, and that data of interest could be collected. We also demonstrated that the intervention resulted in the expected fluid-sparing effect vs. usual care.
- Feasibility findings from the SQUEEZE pilot trial supported proceeding to the large multicenter phase SQUEEZE trial with only minor changes to the protocol.

## Background

Fluid resuscitation has long been a cornerstone of septic shock resuscitation [1, 2]. The 2009 Surviving Sepsis guidelines recommended early and aggressive fluid resuscitation, including successive fluid boluses of 20 mL/kg up to and over 60 mL/kg, followed by initiation of vasoactive medication support for ongoing shock [1]. The Fluid Expansion as Supportive Therapy (FEAST) trial published in 2011 prompted significant interest in guideline recommendations for fluid bolus therapy after demonstrating an increased risk of mortality in children treated with bolus fluids [3]. Emerging observational evidence also began to link fluid overload with an increased risk of morbidity and mortality in adults and children [4–6]. This in conjunction with a lack of randomized controlled trial (RCT) evidence in high-income countries raised the important question of whether fluid boluses were helpful or harmful in children with septic shock with access to advanced critical care [7].

We embarked on the SQUEEZE trial research program to answer this question. We also sought to leverage trial resources to investigate the prognostic value of plasma cell-free deoxyribonucleic acid (cfDNA) levels in children as this biomarker has been demonstrated to be an indicator of poor outcome in adult ICU patients with sepsis [8, 9]. When we began this work in 2013, it was clear that equipoise did not exist for randomizing children to no bolus vs. fluid bolus therapy because isotonic fluid resuscitation remained the standard of care. The evidence for the optimal timing of initiation of vasoactive medications relative to fluid boluses for septic shock resuscitation was also unclear [1]. We therefore planned to investigate a fluid-sparing strategy consisting of restriction of fluid bolus therapy vs. usual care of liberal fluid resuscitation in conjunction with earlier initiation and preferential escalation of vasoactive medication(s) in the fluid-sparing group. We recognized that it was important to enable enrollment prior to pediatric intensive care unit (PICU) admission, that the use of an exception to consent process would be required, and that many potential pitfalls could derail our trial. We determined a pilot RCT was needed to evaluate the feasibility of the SQUEEZE trial protocol and hypothesized that the multicenter SQUEEZE trial, including a nested biomarker-based translational study, was feasible to conduct.

## Materials and methods

The trial design was a pragmatic, two-arm, open-label, prospective pilot RCT. Approval for single-center study conduct was granted by the Hamilton Integrated Research Ethics Board (HIREB) on June 4, 2013 (Project ID: 13–295), with an amendment allowing the addition of an external site. As a trial which enrolled participants experiencing an individual medical emergency, the research ethics board approved protocol included the use of an exception to consent (deferred consent) process as supported by the Canadian Tri-council policy statement guidelines and the Declaration of Helsinki [10]. A summary of protocol versions and amendments is provided (Table S1). The trial was prospectively registered on ClinicalTrials.gov on October 23, 2013 [NCT01973907] prior to the enrollment of the first participant. We prepared the study protocol following the Standard Protocol Items: Recommendations for Interventional Trials (SPIRIT Guidelines) [11, 12]. Our protocol was published in *Trials* where information including the SPIRIT checklist, World Health Organization Trial Registration Data Set, and the schedule of enrollment, interventions, and assessments can be accessed [13]. An extended CONSORT checklist for pilot and feasibility trials is provided (Appendix S1) [14].

The study was promoted to pediatric emergency department (PED) and PICU staff physicians, nurses, and clinical trainees who assisted with the timely identification of potentially eligible patients. Patients were screened and enrolled 24 h/day, 7 days per week by the SQUEEZE trial research assistant or one of the investigators. We enrolled children with suspected or confirmed septic shock and a need for ongoing resuscitation presenting from various locations within a pediatric tertiary care center. This included children 29 days to <18 years of age presenting via the PED, inpatient wards (medical emergency team (MET) activations), and the PICU. A minimum of 40 mL/kg (2 L for children >50 kg) of isotonic fluid bolus therapy within the previous 6 h and ongoing signs of shock were required for inclusion. Full inclusion and exclusion criteria are previously published and presented below [13].

SQUEEZE pilot trial eligibility criteria

**Table:** 

Inclusion criteria	Exclusion criteria
1. Age 29 days to <18 years	1. Patient admitted to the neonatal intensive care unit (NICU)
2a) Persistent signs of shock defined as one or more of: i) Vasoactive medication dependence^a^ ii) Hypotension (systolic and/or mean blood pressure <5th percentile for age)^b^ iii) Abnormal perfusion^c^	2. Full active resuscitative treatment not within the goals of care
2b) Suspected or confirmed septic shock	3. Shock secondary to causes other than sepsis (i.e., obvious signs of cardiogenic shock, anaphylactic shock, hemorrhagic shock, spinal shock)
2c) Fluid resuscitation threshold met. Patient has received within the previous 6 h a minimum of: i) 40 mL/kg of isotonic crystalloid^d^ and/or colloid^e^ as IV fluid bolus therapy for participants <50 kg or ii) 2 L of isotonic crystalloid^d^ and/or colloid^e^ as IV fluid bolus therapy for participants ≥50 kg	4. Patients requiring resuscitation in the operating room or post anesthetic care unit
3. Fluid refractory septic shock as defined by the presence of 2a, 2b, and 2c	5. Previous enrollment in this trial, where known by the research team

^a^Vasoactive medications needed for hemodynamic support, including any of dopamine, dobutamine, epinephrine, norepinephrine, milrinone, phenylephrine, and vasopressin

^b^Guidance for hypotension based on Pediatric Advanced Life Support parameters for the 5th percentile for age

^c^Abnormal perfusion requires the presence of 2 or more of abnormal capillary refill (CR < 1 s (flash) or CR ≥ 3 s (delayed)), tachycardia (heart rate > 95th percentile for age), decreased level of consciousness, or decreased urine output

^d^0.9% normal saline or Ringer’s lactate

^e^5% albumin

To identify eligible non-enrolled patients, we screened the daily PICU census and MET activation records. Eligible patients were randomized using a telephone-accessible third-party computer-based process. The allocation sequence was computer-generated and prepared by the Biostatistics Unit using a schema of simple randomization with no stratification or blocking. The allocation sequence was kept secret from and inaccessible by the investigators.

**Fig. 1:**
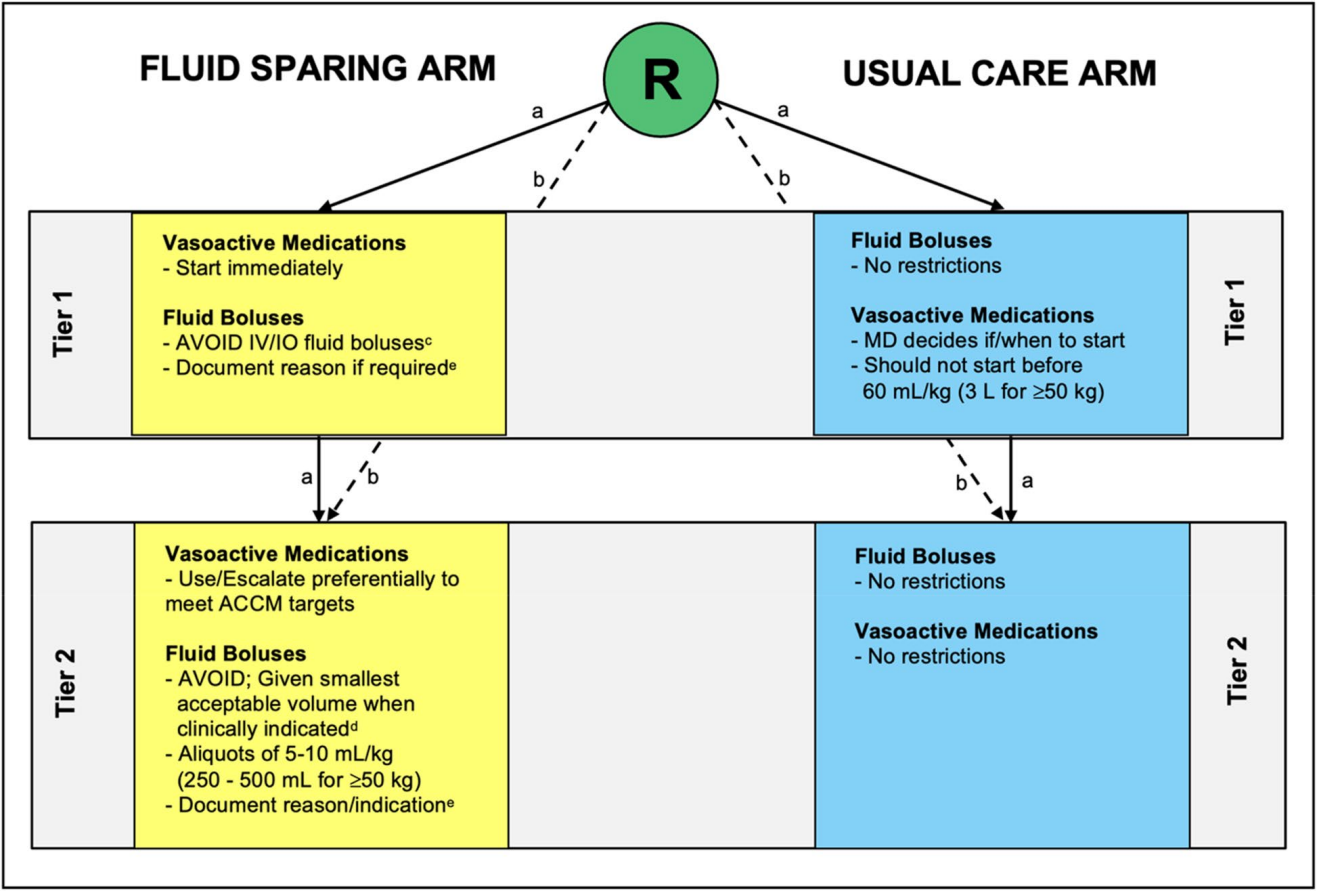
Fluid-sparing (intervention) and usual care (control) management strategies. *This work is licensed under CC BY-NC 4.0 creativecommons.org/licenses/by-nc/4.0. Reproduced with permission from the original source file authored by Parker MJ, available at: http://hdl.handle.net/11375/29921 [16]. Legend: **a** Participants who are randomized prior to initiation of any vasoactive medication infusions begin the assigned allocation in tier 1. Tier 1 contains instructions specific to the situation of a participant not being treated with vasoactive medications. A participant may never exit tier 1. If vasoactive medications are commenced, the treatment instructions shift to those described for tier 2. **b** Participants who are randomized when already receiving a vasoactive medication infusion(s) begin the assigned allocation in tier 2. Tier 1 is bypassed in such situations. **c** Healthcare providers are requested to avoid giving any further fluid boluses. Valid reasons to give a small volume fluid bolus, as noted on the bedside study algorithm, include (i) clinically unacceptable delay in the ability to start a vasoactive medication infusion (e.g., nursing staff need to prepare the infusion) and (ii) documented intravascular hypovolemia (based on clinician assessment). **d** Healthcare providers are requested to avoid giving any further fluid boluses. Valid reason to give a small volume fluid bolus, as noted on the bedside study algorithm, includes (i) documented intravascular hypovolemia (based on clinician assessment). **e** Healthcare provider justification for administration of any fluid bolus to a participant in the fluid-sparing group is to be documented on the fluid-sparing bolus record contained within the study package (fluid-sparing group only). Abbreviations: R, randomization; IV, intravenous; IO, intraosseous; mL, milliliters; kg, kilograms; MD, medical doctor/physician

Following randomization, the group assignment and initial instructions were communicated by the research team to the healthcare team managing the participant. Brief verbal instructions on how to implement the intervention were provided, including direction to obtain a SQUEEZE study package labeled with the group assignment. A detailed description of the intervention is presented elsewhere [13]. In brief, the intervention had two tiers, with each tier providing instructions for vasoactive medication(s) use and fluid bolus therapy (Fig. 1). For both groups, study packages included a copy of the ACCM hemodynamic goals and the surviving sepsis guideline to promote adherence to best practices for aspects of patient care not impacted by the intervention [15]. A group-specific one-page flow diagram provided instructions on how to implement the assigned treatment. Study signs noting the treatment assignment were placed on the medical record and in the patient’s room (at the head of the bed, tag on IV pump) as visible prompts for healthcare team members. For the fluid-sparing arm only, a fluid bolus record was provided to document provider justification for any administered fluid bolus(es). The assigned treatment was continued until shock was determined to be reversed. For the nested translational study (named SQUEEZE-D), blood samples were requested at two timepoints: sample A (baseline, within 6 h of randomization) and sample B (24–48 h following randomization). Samples were collected in a citrated plasma tube (CPT), spun, and stored within 1 h of collection at −80 °C for later measurement of cell-free DNA (cfDNA).

The purpose of conducting a pilot trial is to assess process, resource, management, and scientific aspects of feasibility before embarking on a larger scale trial [17]. The SQUEEZE pilot trial co-primary outcomes included (i) the ability to enroll participants and (ii) initiation of study procedures within 1 h of randomization. We defined a priori the pass threshold for formal evaluation of protocol feasibility as the ability to enroll ≥2 participants per month (per site per month if a second site is added). Due to the multifaceted nature of the intervention, the timing of initiation of study procedures was based on the time of initiation of the applicable treatment algorithm, which was recorded directly on the study package when implemented. Secondary outcomes included the appropriateness of eligibility criteria, the ability to collect clinical outcomes, and data related to study process, resource, and management aspects of feasibility. This included the feasibility of obtaining Ultrasonic Cardiac Output Monitor (USCOM™) assessments of cardiac indices at specified timepoints. The primary feasibility outcome for the nested translational study (named SQUEEZE-D) was to determine the proportion of SQUEEZE participants for whom plasma levels of cfDNA could be described [8, 9]. SQUEEZE-D secondary outcomes included the availability of required samples, as well as study process, resource, and management aspects of feasibility impacting specimen acquisition and testing [13].

Demographic and clinical outcome data for participants were obtained from the medical record. Data were abstracted by trained research staff or one of the investigators and recorded on a paper data collection form for subsequent entry into the electronic REDCap Case Report Form (CRF) [18]. SQUEEZE-D specimen analysis results were retained in a secure file at the Thrombosis and Atherosclerosis Research Institute (TaARI) [13]. In accordance with Canadian research ethics policy, our protocol specified that data collected until consent decline would be retained to minimize the risk of bias. Participants or their substitute decision-makers (SDM) who declined the intervention were asked for permission to continue follow-up for data collection. Consent for SQUEEZE-D participation specifically as it pertained to the use of biological specimens was documented within the same consent form.

We set the sample size for this pilot trial at 50 participants (25 per arm) as this is sufficient to evaluate feasibility [17, 19]. We did not base our sample size on a sample size calculation as this is not required for pilot studies. Considering both the fluid-sparing and usual care treatment strategies fell within the broad scope of Surviving Sepsis treatment recommendations, study participation was deemed minimal risk. Many clinical outcomes in the trial were categorized as adverse outcomes as is common in critical care research [20]. Serious adverse events (SAEs) were defined in accordance with published REB guidance [21]. We planned to report SAEs to the REB and to monitor these at the trial steering committee. We did not form a data safety and monitoring board (DSMB) for this pilot trial considering the minimal risk attributable to study participation and that study duration was too short for the DSMB process [22]. There were no stopping rules or planned interim analysis.

Clinical outcomes included in the trial were selected based on clinical importance and physiologic rationale. These included adverse outcomes potentially attributable to fluid overload as well as adverse outcomes potentially attributable to vasoactive medication use. The rationale for fluid bolus administration in the setting of shock is based on the Frank-Starling principle, whereby an increase in preload following fluid bolus delivery should result in an increase in stroke volume (to a point) [23]. Vasoactive medications (specifically inotropes) should increase the slope of a Frank-Starling curve through increased cardiac contractility, resulting in a greater stroke volume where preload is held constant. However, when preload is inadequate, as may be the case in the setting of septic shock induced vasoplegia, increasing vasoactive support without restoring adequate preload will not effectively treat ongoing shock when cardiac filling is inadequate. For this reason, we identified time to shock reversal a priori as the preferred primary outcome for the main trial. We defined time to shock reversal as the time in hours from randomization until shock was confirmed to be reversed in the absence of ongoing vasoactive medication or mechanical circulatory support (extracorporeal life support). A more detailed description of the time to shock reversal outcome is provided elsewhere [13]. In considering alternative primary outcomes for the main trial, the main contender was the Pediatric Logistic Organ Dysfunction (PELOD-2) score, a measure of the severity of organ dysfunction in children [24]. From an explanatory perspective, we considered this outcome less directly impacted by the physiology the SQUEEZE intervention aimed to address, and it was therefore considered to be our fallback option. Given the low expected mortality rate in pediatric compared to adult septic shock, we considered mortality to be neither a feasible nor appropriate primary outcome for the main trial.

The statistical analysis plan is described within our published pilot trial protocol and included descriptive summary measures for the reporting of baseline characteristics and outcome variables (primary and secondary) [13]. This includes mean (SD) or median (Q1, Q3) for continuous variables and *n* (%) for categorical variables. We prespecified the reporting of feasibility outcomes as descriptive estimates with 95% confidence intervals. The enrollment rate was estimated by dividing the number of participants enrolled by the months of recruitment. Assessment of scientific aspects of feasibility included sample size calculations for the planned multicenter trial. We arranged for an independent statistician to perform the sample size calculation so that we could remain blinded to clinical outcome estimates. We planned to include pilot trial participants in the larger multicenter trial, if feasibility was confirmed, and as such we did not plan to analyze or report clinical outcomes beyond assessment for completeness. For SQUEEZE-D, the translational biosample analysis results were similarly not further analyzed, with reporting planned with main trial results. We specified adherence to the Consolidated Standards of Reporting Trials (CONSORT) extension guidelines for the reporting and analysis of pilot and feasibility RCTs and to have a statistician conduct the analyses using SAS (Cary, NC, USA).

## Results

**Fig. 2:**
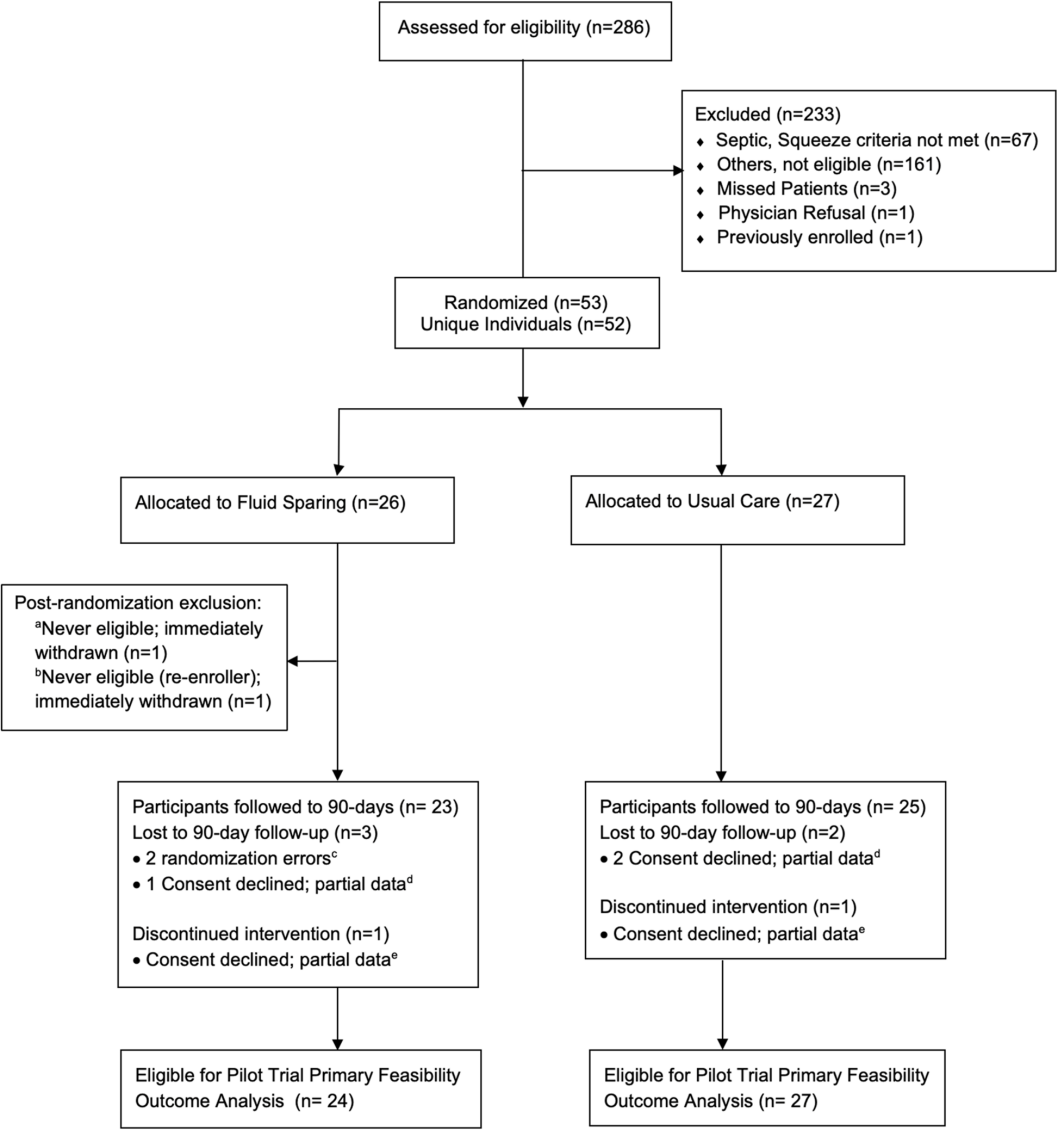
SQUEEZE pilot trial CONSORT flow diagram. Legend: **a** This was an early randomization error shortly after recruitment began. An eager research team member misapplied the screening criteria and randomized a stable ward patient. Immediately recognized; the patient did not receive the intervention and was excluded. No other data is available. **b** This randomization error occurred when a research team member failed to check the log of previously enrolled patients. The error was quickly recognized; the patient did not receive the intervention and was excluded. The first enrollment was retained. **c** The details of these randomization errors^a,b^ are noted above. **d** Due to the exception to consent alternative consent model, participants enrolled may decline consent post-randomization. In such instances, data is retained up until the point of consent decline as described in our protocol. **e** The intervention was only discontinued if it was still being applied at the time when consent was declined

The pilot trial was conducted at McMaster Children’s Hospital, a pediatric tertiary care center in Hamilton, Canada. Participants were enrolled from January 6, 2014, to June 3, 2016, with follow-up to 90 days. The process of flow through the study is illustrated in Fig. 2 in accordance with CONSORT guidelines [25, 26]. At a single center, there were 53 randomization occurrences in 52 unique individuals. This included two randomization errors, with one involving a previously enrolled participant. The analysis population included 51 participants, with 24 in the fluid-sparing and 27 in the usual care group. Consent for ongoing participation was obtained for 48/51 (94%) participants. Baseline characteristics of study participants are presented in Table 1.

**Table 1:** Baseline characteristics

Baseline characteristic	Usual care* (*n* = 27)	Fluid-sparing* (*n* = 24)
Age (months)	94.6 (67.9)	118.9 (58.6)
Male gender	15 (55.6%)	10 (41.7%)
Weight (kg)	31.2 (27.4)	33.9 (22.0)
Admission diagnosis to hospital	27 (100%)	24 (100%)
Sepsis-related	25 (92.6%)	22 (91.7%)
Location of patient screened eligible		
Emergency department	7 (25.9%)	15 (62.5%)
Hospital ward	7 (25.9%)	2 (8.3%)
PICU	13 (48.1)	7 (29.2%)
Previous medical comorbidities	23 (85.2%)	17 (70.8%)
Neurological	10 (43.5%)	11 (64.7%)
Cardiac	5 (21.7%)	2 (11.8%)
Pulmonary	4 (17.4%)	4 (23.5%)
Malignancy	5 (21.7%)	4 (23.5%)
Genetic/hereditary disorder	8 (34.8%)	6 (35.3%)
Other	18 (78.3)	16 (94.1%)
PRISM III score Mean (SD)	10.8 (7.6)	14.5 (9.7)
Heart rate (beats per minute)	143.5 (26.7)	134.3 (27.3)
Systolic blood pressure (mm Hg)	91.8 (13.9)	87.0 (14.6)
Mean blood pressure (mm Hg)	67.0 (10.6); (*n* = 14)	59.7 (8.6); (*n* = 11)
Capillary refill time (seconds)	3 (2, 3); (*n* = 20)	3 (2, 4); (*n* = 16)
Radial pulse quality		
Normal	18 (66.7%)	10 (41.7%)
Weak or thready	4 (14.8%)	5 (20.8%)
Bounding	2 (7.4%)	2 (8.3%)
Not available (not documented)	3 (11.1%)	7 (29.2%)
Mental status altered from baseline		
Yes	18 (66.7%)	17 (70.8%)
Respiratory rate (breaths per minute)	30 (22, 40)	22 (19.5, 31.5)
SpO2 (percent)	97.0 (95.0, 98.5)	98 (97, 99.3)
Body temperature (degrees Celsius)	37.6 (36.8, 38.4); (*n* = 27)	37.2 (36.9, 37.6); (*n* = 23)
pH	7.34 (7.25, 7.39); (*n* = 23)	7.32 (7.24, 7.41); (*n* = 21)
Lactate (mmol/L)	2.0 (1.5, 3.7); (*n* = 21)	2.9 (1.8, 4.0); (*n* = 19)
Bicarbonate (mmol/L)	22 (17, 24); (*n* = 24)	20 (16, 24); (*n* = 22)
Glucose (mmol/L)	6.6 (5.0, 7.3); (*n* = 21)	6.4 (4.8, 12.4); (*n* = 19)
Potassium (mmol/L)	3.9 (3.3, 4.3); (*n* = 24)	3.7 (3.4, 4.1); (*n* = 21)

*Estimates are mean (SD) or median (Q1, Q3) for continuous variables and *n* (%) for categorical variables. Variable is available for group *n* unless otherwise noted

*PICU* pediatric intensive care unit, *PRISM* Pediatric Risk of Mortality, *SD* standard deviation, *kg* kilograms, *mm Hg* millimeter of mercury, *IQR* interquartile range, *SpO2* oxygen saturation

**Fig. 3:**
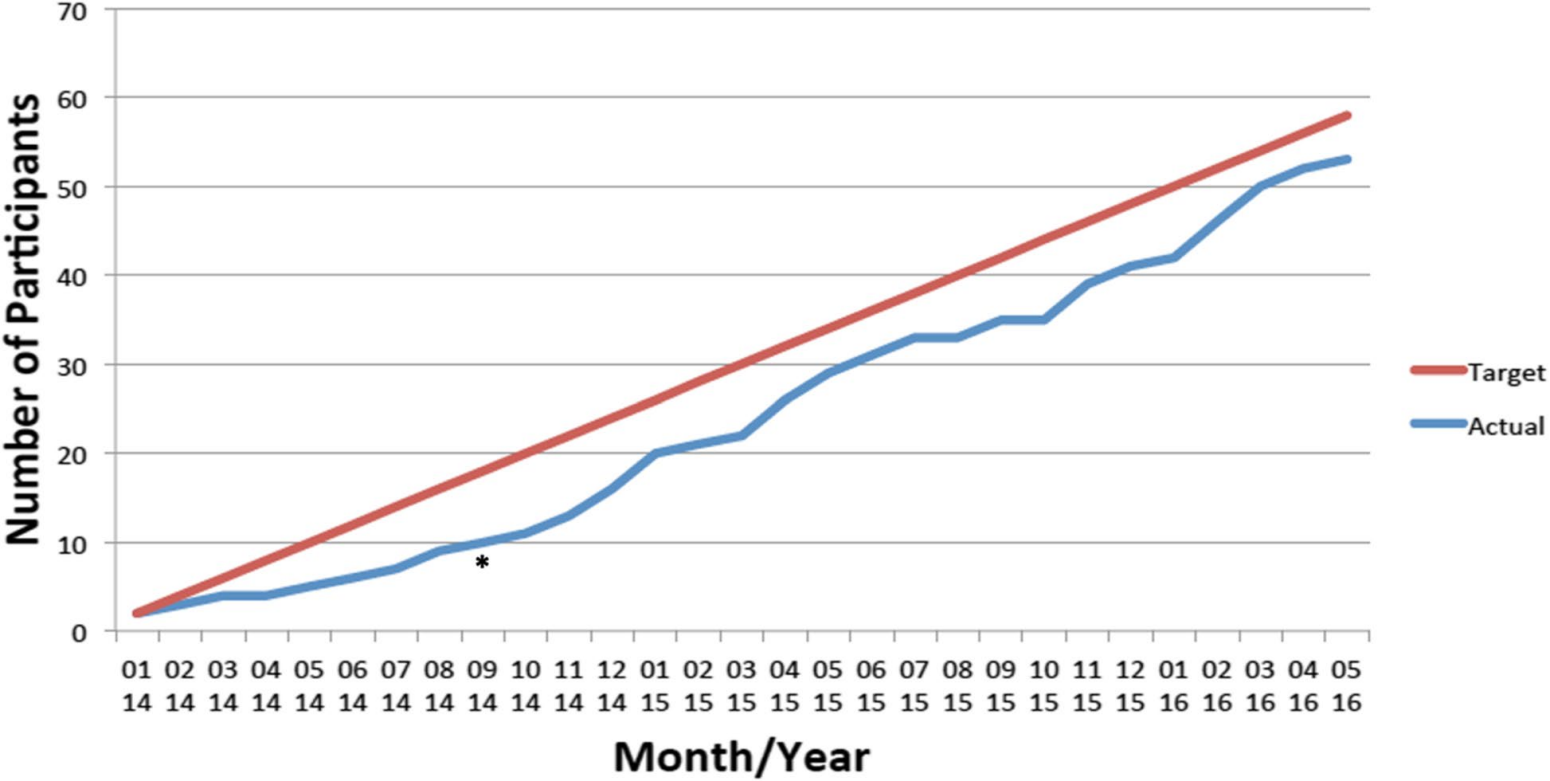
SQUEEZE pilot trial recruitment. Legend: On the y-axis of the figure is the cumulative number of participants randomized into the trial. On the x-axis are the month and year of recruitment. Asterisk (*) denotes timing of implementation of a protocol amendment to an inclusion criteria in September 2014. The amendment lengthened the time window from 2 to 6 h within which the minimum volume of fluid bolus therapy required to meet eligibility criteria was received

SQUEEZE pilot trial feasibility outcomes are presented in Table 2. The enrollment rate was estimated at 1.8 (51/29); 95% C.I. 1.3–2.2 participants/month, which met our feasibility criteria. Importantly, we implemented an early amendment to one of the inclusion criteria based on our experience during the first 6 months of recruitment. We identified that our initial time window of 2 h for the minimum fluid administration criteria to be met was too short and either delayed participant entry into the trial or resulted in the exclusion of patients we believed should be included. A protocol amendment increasing this timeframe from 2 to 6 h was implemented following REB approval in September of 2014 and appeared to impact the slope of recruitment for the remainder of the trial (Fig. 3). The post-amendment recruitment rate was estimated at 2.0 (41/21); 95% C.I. 1.4–2.6 participants/month.

**Table 2:** SQUEEZE pilot trial feasibility outcomes

Pilot trial outcomes	Analysis result *N* (%) or count per month; 95% C.I.	Criteria for success of feasibility
SQUEEZE		
SQUEEZE primary outcomes		
1.1 Participant enrollment rate		Enrollment ≥2 participants/month
Entire study period^a^ (participants/month)	51/29 (1.8; C.I. 1.3–2.3)	
Following early amendment^b^ (participants/month)	41/21 (2.0; C.I. 1.4–2.6)	
1.2 Consent rate for continued participation	48/51 (94.1%), C.I. 83.8–98.8%	N/A
1.3 Missed eligible patients (patients/month)	3/29 (0.1; C.I. 0.0–0.3)	N/A
2. Protocol adherence		
Ability to initiate study within 1 h of randomization	49/51 (96.1%), C.I. 86.5–99.5%	N/A
Median (IQR) (minutes)	8 (5, 15)	
Range (minutes)	2–177	
SQUEEZE secondary outcomes		
1. Appropriateness of eligibility criteria as evidenced by ability to enroll in a timely manner	Yes, following early amendment of one inclusion criterion	Pass based on ability to enroll
2. Completeness of clinical outcomes of interest	See supplemental Table S2	N/A
3. Considerations related to study process feasibility	See supplemental Table S3	N/A
4. Considerations related to study resource feasibility	See supplemental Table S4	N/A
5. Considerations related to study management aspects of feasibility	See supplemental Table S5	N/A
SQUEEZE-D		
SQUEEZE-D primary outcome		
Proportion of SQUEEZE participants for whom cfDNA can be described for both timepoint A^c^ and B^d^	22/51 (43.1%), CI: 29.4–57.8%	N/A
SQUEEZE-D secondary outcomes		
Availability of the required samples from patients enrolled in SQUEEZE		N/A
Both timepoint A^c^ and B^d^	22/51 (43.1%), CI: 29.4–57.8%	
Timepoint A^c^	33/51 (64.7%; CI: 50.0–77.6%	
Timepoint B^d^	25/51 (49.0%), CI: 34.8–63.4%	
SQUEEZE-D process, resource, and management aspects of feasibility	See supplemental Table S6	N/A

^a^Enrollment rate over the entire 29-month study period

^b^Enrollment rate calculated for the 21-month period subsequent to implementation of amendment to an inclusion criteria

^c^Timepoint A is at baseline (within 6 h following randomization)

^d^Timepoint B is between 24 and 48 h following randomization; interquartile range (IQR); cell-free deoxyribonucleic acid (cfDNA)

The median (IQR) time to initiate study procedures was 8 (5, 15) minutes, and implemented within 1 h of randomization in 49/51 (96.1%; 95% C.I. 86.5–99.5%). In the two instances where this was not achieved, one involved a delay in reaching a member of the healthcare team, while in the other, study package implementation was delayed. The completeness of hemodynamic and clinical outcome data of interest for the full trial is presented in Table S2. Additional supplementary tables present secondary outcome findings related to study process (Table S3), resource (Table S4), and management (Table S5) aspects of feasibility and for SQUEEZE-D (Table S6). Our ability to collect pre-randomization (Table S7) and post-randomization (Table S8) data describing fluid and blood product intake and output, and hemodynamic descriptive data (Table S9) are also provided. Completeness of pre- and post-randomization descriptive data for culture results, antimicrobial therapy, and laboratory data is presented in Tables S10 and S11.

**Fig. 4:**
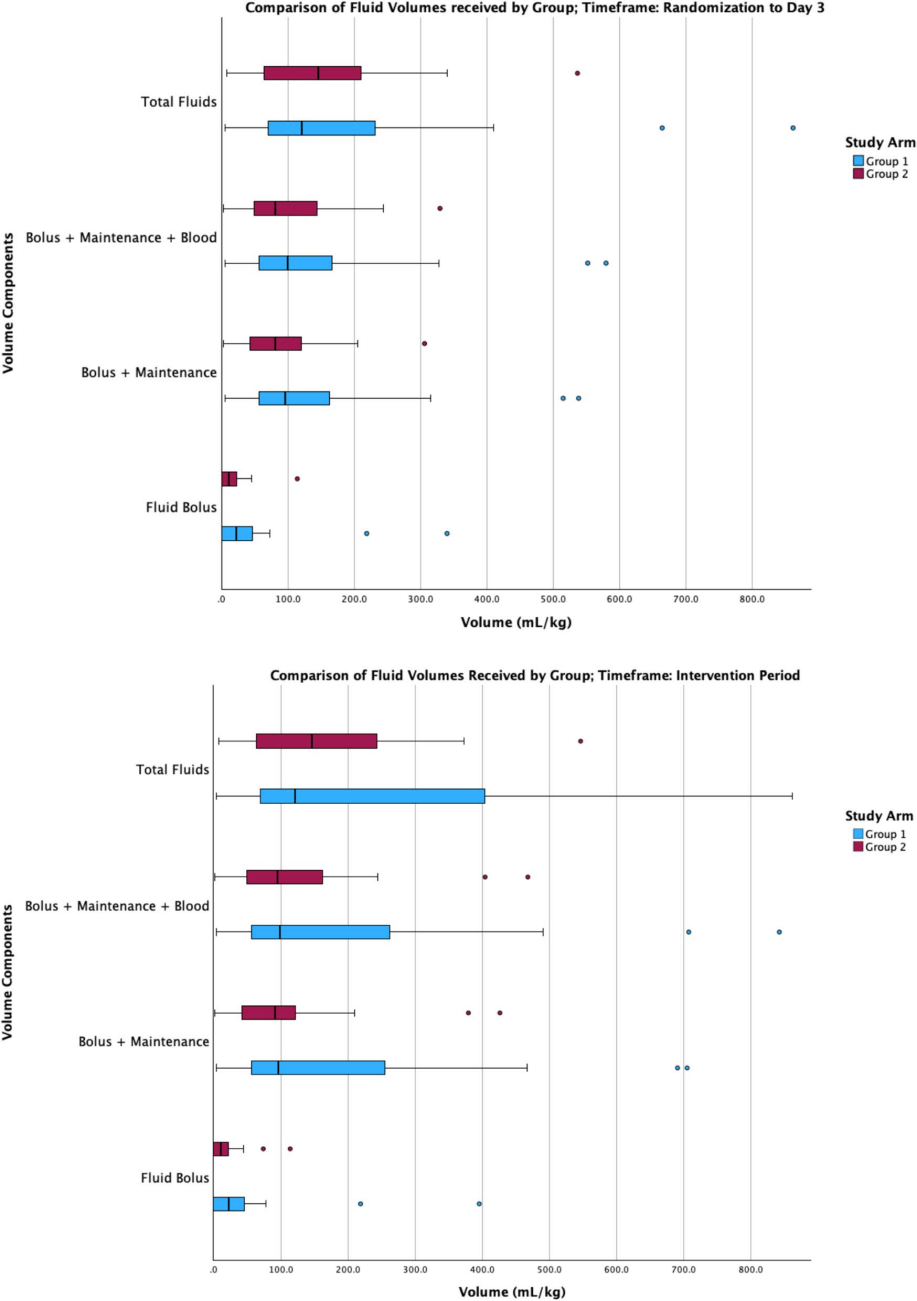
Between group separation in fluid volume received reported by volume components. Legend: Two-panel clustered boxplot figure displaying fluid volume received by volume component and study arm for two different timeframes. The y-axis of the two figures displays the fluid volume components represented as distinct clusters: Fluid Bolus includes isotonic fluid boluses only, Bolus + Maintenance includes isotonic fluid boluses plus maintenance fluids, Bolus + Maintenance + Blood includes isotonic fluid boluses plus maintenance fluids plus blood products, Total Fluids includes all fluid intake. The x-axis of the two figures displays mean fluid volume received (mL/kg). In both figures, group 1 is usual care (control) and group 2 is fluid-sparing (intervention). Boxplot whiskers denote 1.5 times the interquartile range. On the bottom figure, for “Total Fluids,” 4 outliers (2 in group 1 and 2 in group 2) are not shown. In the figure on the top, the timeframe is defined as the first six 12-h periods (based on nursing shifts) following randomization, with the first shift a partial shift. In the figure on the bottom, the intervention period is defined as the period from randomization until shock is reversed for each participant

For scientific aspects of feasibility, there were no adverse events or SAEs. Protocol deviations, violations, suspensions, and withdrawals (reported in Table S3) are relevant from a scientific perspective due to their potential to impact the effectiveness of the intervention and to introduce risk of bias in the case of withdrawals. We assessed the effectiveness of the intervention to spare fluid at a single timepoint given the open-label design of the trial. These interim results as presented in our 2016 application to CIHR for large multicenter trial funding are presented in Fig. S1. Exploration of the finalized pilot trial dataset revealed that data for fluid volumes administered were unlikely to be normally distributed based on Shapiro-Wilk testing for normality. As such, fluid volume data are shown in Fig. 4 in clustered boxplot format. Between randomization and day 3, the median (interquartile range) volume of isotonic bolus fluids administered was 22.1 (0.0, 48.7); (*n* = 27) mL/kg in group 1 (usual care) compared to 10.3 (0.0, 22.7); (*n* = 24) mL/kg in group 2 (fluid-sparing). For the entire period across which the intervention was applied, the median (interquartile range) volume of isotonic bolus fluids administered was 22.1 (0.0, 48.7); (*n* = 27) mL/kg in group 1 (usual care) compared to 10.3 (0.0, 22.7); (*n* = 24) mL/kg in group 2 (fluid-sparing). Additional descriptive data for fluid administration by volume component are presented in Table S12.

Sample size calculations based on the outcome of time to shock reversal were prepared by an independent statistician and shared with the investigators as a sample size table [27]. Using the standard deviation (1.2) observed in the pilot trial and an adjustment rate of +12%, it was determined that 400 participants (200 per arm) were required for a multicenter trial to detect an estimated 30% difference in the time to shock reversal based on a two-sided *t*-test of the null hypothesis that there is no difference between groups (based on geometric mean), with type 1 error (*α*) at 0.05 and power (*β*) at 80%. We considered this difference minimally clinically meaningful as this corresponded to approximately one 12-h nursing shift. It is important to note the use of the geometric mean for statistical testing. Use of geometric means for between-group comparisons is appropriate when data may be skewed—a situation where use of group means would otherwise be inappropriate. An alternative approach in the setting of skewed outcome data could be the comparison of group medians. While survival analysis was considered for the main trial, the sample size required for this analysis approach was prohibitive.

## Discussion

We completed a single-center pilot randomized controlled trial evaluating a fluid-sparing intervention vs. usual care in children with a clinical diagnosis of septic shock and a need for ongoing resuscitation. The main finding of our study was that the protocol was feasible to conduct and this supported progression to a larger, multicenter trial based on our ability to enroll participants and rapidly implement study procedures. Our second main finding was that adherence to the protocol was effective in achieving between-group separation in the volume of fluid received. Thirdly, our experience with study procedures and data collection informed the refinement of the protocol and data collection plan for the main trial. Finally, our approach to operationalizing the exception to consent process used in the trial worked well and was generally well accepted.

In designing the SQUEEZE protocol, our aim was for the study to be pragmatic so that our findings would be widely generalizable. We therefore recruited from various locations within the hospital including the PED, inpatient wards, and PICU. We decided not to recruit from community hospital sites due to the rarity of pediatric septic shock in conjunction with the expense and challenges of training and maintaining the engagement of community physicians. Inwald et al. attempted recruitment of a similar population from community hospitals during this timeframe and deemed their protocol infeasible [28]. We decided against recruitment in the prehospital environment for similar reasons. Patients critically ill enough to require transfer to tertiary care retained the opportunity to be enrolled if they met eligibility criteria upon arrival. This was an important feature of our protocol which supported enrollment into either tier 1 or tier 2 of the intervention. We considered this analogous to the real-world setting where clinicians implement a guideline according to a patient’s clinical status when they assume care.

Another important aspect of our eligibility criteria was the minimum required volume of fluid bolus therapy and the timeframe within which this was received. We selected a minimum volume of 40 mL/kg (2 L for >50 kg) which corresponded to 2 fluid boluses because we believed this was necessary to select children at risk for relatively rare outcomes of interest. In comparison with two other pilot trials investigating interventions to spare fluid, we succeeded in enrolling sicker children, as evidenced by participant PRISM III scores [28, 29].

Adherence to the SQUEEZE protocol was effective in achieving a visible fluid-sparing effect as shown in Fig. 4. This was sustained for at least 72 h, which we attribute to our intervention being applied until shock reversal was confirmed. In designing the SQUEEZE intervention, we believed that early initiation of vasoactive medication(s), while important, was insufficient to result in a meaningful difference in fluid volume received over an extended period of time. Prioritized use of vasoactive medications provided an alternative strategy to target recommended hemodynamic endpoints while avoiding or otherwise limiting further fluid bolus therapy. Protocol deviations occurred in a minority of participants and did not compromise between-group separation in the volume of fluid received.

Testing our protocol and data collection plan provided significant learning opportunities. One unanticipated finding was that the telephone-accessible randomization system required 24/7 was vulnerable to power outages. We therefore shifted to REDCap-based randomization for the main trial. We also learned that having the study pager linked to the MET paging waterfall resulted in an excessive number of calls and high screening workload with the majority of these patients ineligible and so this was abandoned. USCOM assessments could not be reliably completed and were eliminated. Finally, our experience allowed us to refine our data collection plan for the main phase of the trial. Radial pulse quality was eliminated as this was not documented by nursing staff. We shifted from PRISM III to PRISM IV due to its improved reliability [30]. Acute kidney injury was added as an outcome, as its exclusion from the pilot trial CRF was an oversight.

The SQUEEZE pilot trial was the first study in Canada to enroll children into a RCT using an exception to consent process. This alternative consent model provides a temporary waiver of consent to enable research in individual medical emergencies and allows enrollment, initiation of study procedures, and data collection until such time as full informed consent (and assent as applicable) can be completed. We implemented this using a practical approach of providing a one-page notice of enrollment to SDMs as early as possible to inform them of their child’s enrollment. This was followed by at minimum daily research team contact with the participant’s healthcare team to determine the most appropriate time to approach the SDMs for full informed consent discussions. Our approach was generally well accepted, and this has informed other trials since SQUEEZE began [31].

Our pilot trial had several limitations. We expected some post-randomization exit due to consent decline and that some outcomes for these participants may be unavailable which could compromise study validity [32]. We did not anticipate randomization errors; however, these are well-described and, in retrospect, not surprising in a fast-paced resuscitation trial [33]. Learnings from our experience with the pilot trial protocol were useful to inform site training for the large multicenter phase of the trial. Overall, our loss to follow-up rate was low. The very low number of missed patients reflects the high degree of interest in the trial and few gaps in research team on-call coverage, which we expected may not be replicable at external sites. We did attempt to add a second site to evaluate external feasibility; however, the pilot trial recruitment target was reached before the external site could be added. Finally, our pilot trial took over 2 years to complete, reflecting the challenges of conducting interventional research in critically ill children.

## Conclusions

We determined that the multicenter SQUEEZE trial was feasible to conduct. Pilot trial data were essential to justify funding and inform the conduct of the full-scale multicenter trial which is now complete. We believe the important lessons learned from this pilot trial will be of interest to readers, given the complexities of conducting time-sensitive pediatric resuscitation research.

## Supplementary Information

Supplementary Material 1.

Supplementary Material 2.

## Data Availability

Data and material that support the findings of this study are not openly available due to reasons of sensitivity and are available from the corresponding author upon reasonable request. Data are located in controlled access data storage at McMaster University.
